# The Effect of Doctor-Consumer Interaction on Social Media on Consumers’ Health Behaviors: Cross-Sectional Study

**DOI:** 10.2196/jmir.9003

**Published:** 2018-02-28

**Authors:** Tailai Wu, Zhaohua Deng, Zhanchun Feng, Darrell J Gaskin, Donglan Zhang, Ruoxi Wang

**Affiliations:** ^1^ School of Medicine and Health Management Tongji Medical College Huazhong University of Science and Technology Wuhan China; ^2^ Department of Health Policy and Management Bloomberg School of Public Health Johns Hopkins University Baltimore, MD United States; ^3^ Department of Health Policy and Management College of Public Health University of Georgia Athens, GA United States

**Keywords:** physician patient relationships, health behavior, social media, social theory, psychological theory, medical informatics

## Abstract

**Background:**

Both doctors and consumers have engaged in using social media for health purposes. Social media has changed traditional one-to-one communication between doctors and patients to many-to-many communication between doctors and consumers. However, little is known about the effect of doctor-consumer interaction on consumers’ health behaviors.

**Objective:**

The aim of this study was to investigate how doctor-consumer interaction in social media affects consumers’ health behaviors.

**Methods:**

On the basis of professional-client interaction theory and social cognitive theory, we propose that doctor-consumer interaction can be divided into instrumental interaction and affective interaction. These two types of interactions influence consumers’ health behaviors through declarative knowledge (DK), self-efficacy (SE), and outcome expectancy (OE). To validate our proposed research model, we employed the survey method and developed corresponding measurement instruments for constructs in our research model. A total of 352 valid answers were collected, and partial least square was performed to analyze the data.

**Results:**

Instrumental doctor-consumer interaction was found to influence consumers’ DK (*t*_294_=5.763, *P*<.001), SE (*t*_294_=4.891, *P*<.001), and OE (*t*_294_=7.554, *P*<.001) significantly, whereas affective doctor-consumer interaction also impacted consumers’ DK (*t*_294_=4.025, *P*<.001), SE (*t*_294_=4.775, *P*<.001), and OE (*t*_294_=4.855, *P*<.001). Meanwhile, consumers’ DK (*t*_294_=3.838, *P*<.001), SE (*t*_294_=3.824, *P*<.001), and OE (*t*_294_=2.985, *P*<.01) all significantly affected consumers’ health behaviors. Our mediation analysis showed that consumers’ DK, SE, and OE partially mediated the effect of instrumental interaction on health behaviors, whereas the three mediators fully mediated the effect of affective interaction on health behaviors.

**Conclusions:**

Compared with many intentional intervention programs, doctor-consumer interaction can be treated as a natural cost-effective intervention to promote consumers’ health behaviors. Meanwhile, both instrumental and affective interaction should be highlighted for the best interaction results. DK, SE, and OE are working mechanisms of doctor-consumer interaction.

## Introduction

### Background

Social media is penetrating people’s daily life and influencing their health-related activities. Consumers, patients and nonpatients included, are interacting with health professionals or with each other on social media more often than ever before. In the United States, 81% of adults have social media profiles [1]. In China, the most populous country in the world, the number of social media consumers is estimated to reach 679.19 million by 2021 [2]. A US-based study indicates that nearly one-third of consumers’ health-related activities are conducted through social media and almost two-third of consumers search for information regarding a specific doctor or a health professional using social media [3]. Moreover, almost half of consumers claim that information from social media affects their health-related decisions, and more than half of them trust doctors’ Web-based posts or blogs [4]. Currently, more than 60% of doctors use various forms of social media for personal or professional reasons, and this percentage has been increasing in recent years [5]. Both consumers and doctors have engaged in using social media to disseminate health-related information, and therefore, social media could be an important medium for interactions between doctors and consumers.

Social media are Internet-based applications that build on Web 2.0 techniques to allow the creation and exchange of user-generated content. These applications can replace the traditional one-to-one communication with the many-to-many communication paradigm between patients and doctors [6]. However, doctors’ behaviors on social media may be different from those in the offline context. It may be challenging to apply principles of medical practice for doctors in the social media setting because social media may make them feel less restrained [7]. Besides, social media empowers consumers by providing them with not only the opportunities to interact with many doctors at the same time but also the access to know other consumers with similar interests or experiences [8]. Moreover, the content of interaction may be different in a social media setting. As doctors cannot provide medical diagnosis or treatment using social media directly, the health problems discussed during the interaction may not be acute and serious, consumers may feel less anxious, and therefore, doctors’ affective behaviors may not be as important as they are in the offline context. Hence, the new communication approach, behaviors, and content affect the relationship between consumers and health professionals, which may influence consumers’ health outcomes and well-being [9].

Despite the fact that individuals’ health outcomes of using social media, including health-related emotions, physical conditions, and beliefs, have been well studied, social media’s impact on health behaviors is less understood [10]. Improving health behaviors, such as ceasing smoking, increasing physical activity, keeping a healthy diet, and avoiding overconsumption of alcohol, can substantially lower the risk of dying prematurely [11]. Health behaviors have been found to be correlated with many chronic noninfectious diseases such as diabetes [12], hypertension [13], stroke [14], Alzheimer disease [15], and even cancer [16]. Moreover, unhealthy lifestyle leads to poor health status, obesity [17], depression, anxiety [18], and even poor academic performance [19]. Improving health behaviors at the population level also helps promote health equity in the society [20]. Given the significant impact of health behaviors, policy makers in different countries have taken actions to promote health behaviors. For example, the US Department of Health and Human Services has introduced Healthy People 2020 to promote health behaviors [21]. In the meantime, the State Council of China has set promoting healthy lifestyle among Chinese people as one of the major goals of the Healthy China 2030 Program [22]. Therefore, developing effective interventions to improve health behaviors is very meaningful and contributive. With regard to health behaviors in the social media context, prior literature has shown that several interventions based on social media are effective in changing patients’ behaviors and promoting their health status [23-25]. Nevertheless, the effect of interaction between doctors and consumers on social media on consumers’ health behaviors has not been studied. Thus, our research question is as follows:

How does doctor-consumer interaction on social media influence consumers’ health behaviors?

Overall, we hypothesize that doctor-consumer interaction influences consumers’ health behaviors significantly through some potential pathways. Compared with health promotion interventions using traditional approaches, doctor-consumer interaction on social media could be a low-cost health promotion intervention [26]. Therefore, it is worth evaluating the effect of doctor-consumer interaction and identifying the mechanisms of how it works. To address this question, we ground our research on professional-client interaction theory to conceptualize doctor-consumer interaction in the social media context and social cognitive theory to explore the working mechanisms of doctor-consumer interaction.

### Theoretical Foundation

In this study, we integrate professional-client interaction theory and social cognitive theory to help us understand the effect of doctor-consumer interaction on health literacy. Professional-client interaction theory is mainly used to comprehend doctor-consumer interaction because the subtypes of interaction can be used to describe doctor-consumer interaction, whereas social cognitive theory is used to explore the working mechanisms of doctor-consumer interaction in this study because the interaction can be treated as a learning process.

Professional-client interaction theory claims that physicians’ behaviors toward patients in physician-patient interaction could be classified as instrumental behaviors and affective behaviors [27]. Instrumental behavior is about the content of physicians’ behaviors that focuses on the solution of a health problem, whereas affective behavior is about the mode of physicians’ behaviors that requires physicians to treat patients as a person rather than a case [28]. We argue that the categorization of physicians’ behaviors toward patients in professional-client interaction can be extended to the social media context as doctors can still solve consumers’ health problems and provide emotional support on social media. To contextualize the professional-client interaction in the social media context, we divide doctor-consumer interaction into instrumental interaction and affective interaction [29]. On the basis of instrumental behavior, we define instrumental interaction as a doctor-consumer interaction that focuses on the solution of consumers’ health concern. Toward affective interaction, we define it as the interaction that cares about consumers’ emotions in line with affective behaviors. The effect of instrumental and affective interaction is reasoned and hypothesized in the following sections.

Social cognitive theory, originally labeled as social learning theory, assumes that one learns by observing models’ behaviors and performs their behaviors in the social context [30]. Meanwhile, the maintenance of learned behaviors over time depends on self-regulation and reinforcement. Learned behaviors are results of the dynamic reciprocal interaction among personal, behavioral, and environmental determinants. Furthermore, learned behaviors continue to interact with personal and environmental determinants in the reinforcement process, where beneficial behaviors are repeated and others are avoided. The determinants of learned behaviors can be categorized into 5 categories: outcome expectancy (OE), observational learning, environmental factors, self-regulation, and moral disengagement [31]. Besides, among environmental factors, incentive motivation and facilitation are the 2 main factors [32]. Incentive motivation is a reward or punishment from the environment, whereas facilitation is a resource or tool for facilitating behaviors. In our study, through interacting with doctors on social media, consumers’ health behaviors can be developed in the interaction process because doctors can be the role model or the information source of healthy lifestyle behaviors. Therefore, doctor-consumer interaction can be treated as a learning process and be understood by social cognitive theory. The determinants of learned behaviors in social cognitive theory could be referred to explore the determinants of health behaviors.

### Research Model and Hypotheses

**Figure 1:**
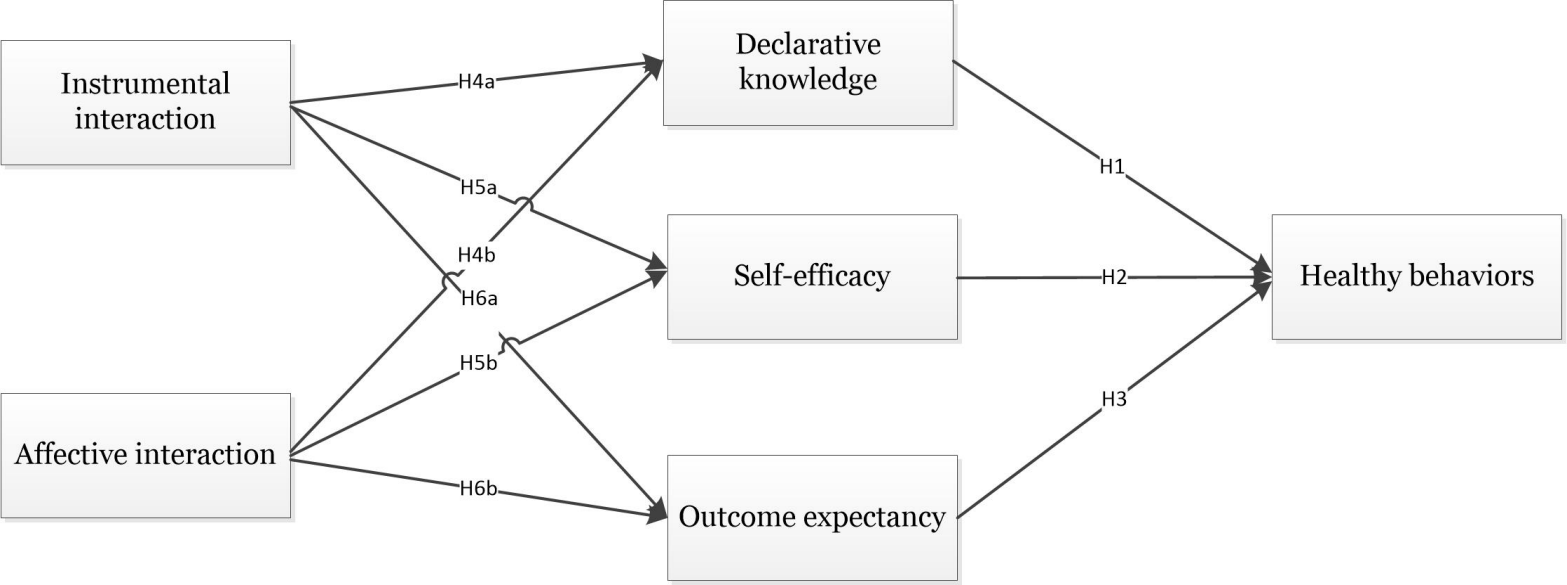
Research model.

According to professional-client theory, we divide doctor-consumer interaction into instrumental interaction and affective interaction. Meanwhile, according to social cognitive theory, we propose that the 2 types of interaction influence consumers’ health behaviors through declarative knowledge (DK), self-efficacy (SE), and OE. The specific hypothetic relationships are depicted in Figure 1.

### Declarative Knowledge, Self-Efficacy, Outcome Expectancy, and Health Behaviors

According to the content of knowledge, knowledge can be classified as declarative knowledge and procedural knowledge [33]. DK is about facts and things that concern the static properties of objects, persons, or events, whereas procedural knowledge is about dynamic skillful actions. For example, information about attributes, facts, and situations is declarative knowledge, whereas procedures for actions or experience are usually referred to as procedural knowledge. Therefore, DK is easy to be communicated and described by verb, whereas procedural knowledge should be acquired in practice. Because consumers only can learn the procedural knowledge when they practice it, DK is more feasible and suitable in our context. Because DK can help people access to the meaning of health behaviors [34], the meaning of health behaviors influences people’s attitudes and their behaviors. Therefore, we can hypothesize the following:

H1: DK positively influences consumers’ health behaviors.

SE is people’s judgment of their capability to perform a specific behavior or task [30]. It has 3 dimensions: magnitude, strength, and generalizability. Magnitude of SE refers to the degree of difficulty to which people believe they can attain a certain kind of behavior, whereas strength of SE is confidence about the judgment. Generalizability reflects the degree to which the judgment can be generalized to different situations. In our context, SE can be consumers’ judgment to master the cognitive and social skills to improve or maintain their health status. As SE can affect people’s level of effort and persistence on a specific behavior according to the dimensions of SE [35], high SE may lead people to put in more effort to do the behaviors and insist on them longer. Hence, we can hypothesize the following:

H2: SE positively influences consumers’ health behaviors.

OE refers to the belief that the expected outcomes are resulted in given behaviors [36]. Three forms of OE exist, including physical form, social form, and self-evaluation form. Physical outcomes include positive and negative effects of behaviors, while the given behaviors may also receive social approval and disapproval. Self-evaluation toward the given behaviors may also be positive or negative [37]. Consumers’ behaviors are regulated by these different forms according to given behaviors’ consequences. In our study, OE is about whether consumers’ interaction with doctors on social media can solve their health concerns or problems. Since people are generally rational, their self-interest behaviors can be regulated by the outcomes of behaviors [38]. Positive outcomes may stimulate people to implement the corresponding behaviors. Therefore, we can hypothesize the following:

H3: OE positively influences consumers’ health behaviors.

### Doctor-Consumer Interaction and Declarative Knowledge

In doctor-consumer interaction, consumers not only have opportunities to ask more questions to doctors but also have access to health information from other sources. Therefore, the doctor-consumer interaction makes consumers acquire health information that can be processed and authenticated to form health knowledge [39]. Given that consumers who interact with doctors on social media can use only Web-based digital tools including texts, pictures, or videos, they cannot make use of the health information on social media directly. Therefore, doctor-consumer interaction can increase consumers’ DK. With regard to 2 types of doctor-consumer interaction, consumers can receive information about their health problems directly in instrumental interaction and get information about dealing with their anxiety in affective interaction [40]. Thus, we can hypothesize the following:

H4a: Instrumental interaction between consumers and doctors on social media positively influences consumers’ DK.

H4b: Affective interaction between consumers and doctors on social media positively influences consumers’ DK.

### Doctor-Consumer Interaction and Self-Efficacy

Four information cues have been proposed to influence the formation of SE: enactive mastery, vicarious experience, verbal persuasion, and emotional arousal [41]. Enactive mastery is people’s performance attainment, whereas vicarious experience is from behavioral models. Verbal persuasion is to make people believe their capability of doing some tasks, whereas emotional arousal is the psychological state that arouses people’s capability. In doctor-consumer interaction, doctors can be the behavioral models who give vicarious experience to consumers and may persuade consumers to develop health behaviors. In our study, toward the relationship between doctor-consumer interaction and SE, vicarious experience and verb persuasion can be the mediating processes. Thus, we can hypothesize the following:

H5a: Instrumental interaction between consumers and doctors on social media positively influences consumers’ SE.

H5b: Affective interaction between consumers and doctors on social media positively influences consumers’ SE.

### Doctor-Consumer Interaction and Outcome Expectancy

Consumers’ OE can also be affected by vicarious experience [42]. Positive learned experience conveys the possible reward of doing specific behaviors and strengthens one’s expectation of positive outcome. In our study, as doctors can be health models and provide support to consumers during the interaction with them, the possibilities of solving consumers’ health problems and performing healthy lifestyle behaviors are increased [43]. Meanwhile, interacting with doctors on social media enforces the social ties between consumers and doctors and helps consumers acquire different kinds of social support from doctors. Thus, we can hypothesize the following:

H6a: Instrumental interaction between consumers and doctors on social media positively influences consumers’ OE.

H6b: Affective interaction between consumers and doctors on social media positively influences consumers’ OE.

## Methods

### Data Collection

Data were collected in China, which has the largest social media market in the world. The Web-based survey was conducted using Zhubajie, which is the biggest crowdsourcing platform in China. Survey announcement was posted in Zhubajie, and registered service providers were invited to fill the questionnaires. In the announcement, we set several requirements to judge whether the answers were qualified. The requirements included using social media, having experiences of interacting with doctors on social media, and filling the questionnaire sincerely, etc. Service providers whose answers met our requirements received a token of appreciation, whereas providers who failed our requirements did not receive the token. Participants also provided informed consent before they filled the questionnaires. After 2 weeks, we obtained a total of 435 responses from Chinese social media consumers who had experienced interactions with doctors on social media.

Because we used Web-based data, several actions were taken to ensure the validity of dataset [44]. To identify the applicable respondents, we set screening questions to check whether the respondents were consumers who interacted with doctors on social media, such as whether participants followed doctors on social media, whether they replied to doctors’ posts on social media, and whether they forwarded doctors’ posts on social media. To avoid responses from experienced survey takers or ones with less attention, we discarded 39 cases that took less than 5 min and checked the cases with missing values or similar values for all questions. To address cheating issues, we did not use the data from respondents who had not correctly responded to the set reverse-coded questions. Thus, we were left with 352 complete and valid responses. In this sample, most of the respondents were in the age group of 25-30 years, were females, possessed a college degree, and were familiar with using social media. This is reasonably consistent with the report of China Internet Network Information Center on demographics of Chinese social media consumers [45]. The specific demographic information of our final sample is summarized in Table 1.

### Measurement Instrument

To validate our research model, we used the survey method in this study. The survey instrument was developed by adapting previously validated scales to the context of our study. Items for affective and instrumental interaction were adapted from Ben-Sira who had studied relevant variables [28]. Items for DK and health behaviors were adapted from the Activity Question Scale, Nutrition Knowledge Scale, and Health Lifestyle Behavior Scale [46]. Items for SE were adapted from the General Self-Efficacy Scale [47] and those for OE were from the Anderson et al study, which had covered OE in other context [48]. A total of 42 items that contain screening questions and demographic questions were presented in the questionnaire. All items were measured on a 5-point Likert scale with anchors ranging from strongly disagree to strongly agree.

As the survey instrument was originally developed in English, we used the back translation method to translate it into Chinese. The English instrument was first translated into Chinese by one of the bilingual authors, TW, whose native language was Chinese. Next, another bilingual author, DZ, back translated the Chinese version into English. The 2 authors then compared the 2 English versions to check for inconsistency, if any. A pretest was conducted on the developed survey instrument by interviewing 8 experts in the area of information systems, medical informatics, and health management and 17 users of social media. We further revised the questionnaire based on the comments and suggestions received. The survey instrument is presented in Multimedia Appendix 1.

### Statistical Analysis

This study employed structural equation modeling using partial least square (PLS) analysis. As the second-generation multivariate causal analysis method, PLS can be applied to complex structural equation models and is less restrictive on sample size than other methods [49,50]. Meanwhile, PLS is suitable for exploratory studies as it aims at theory building rather than theory testing. The analysis was conducted by using SmartPLS 2.0.3M of SmartPLS GmbH in Germany [51].

We analyzed the reliability and validity of measurement instruments using confirmatory factor analysis. As shown in Table 2, all Cronbach alpha and composite reliabilities are above 0.6, thus demonstrating reliability for all constructs [52]. The value of average variance extracted (AVE) of each construct is above 0.5 and items’ loadings are above 0.7, thus demonstrating good convergent validity [52]. On the basis of the results shown in Table 3, the square roots of the AVEs are all greater than the interconstruct correlations, thus demonstrating discriminant validity [53]. Hence, we conclude that the quality of the measurement model is adequate for testing hypothesized relationships.

**Table 1:** Demographic information.

Characteristics		n (%)
**Age in years**		
	<25	122 (34.7)
	25-30	150 (42.6)
	>30	80 (22.7)
**Gender**		
	Male	153 (43.5)
	Female	199 (56.5)
**Education**		
	High school	35 (9.9)
	College	304 (86.4)
	Master’s degree and above	13 (3.7)
**Duration of using social media within a day**		
	<1 hour/day	164 (46.6)
	1-3 hours/day	128 (36.4)
	>3 hours/day	60 (17)
**Experiences of using social media**		
	<1 year	29 (8.2)
	1-5 years	201 (57.1)
	More than 5 years	122 (34.7)

We also examined the possibility of common method bias in our study. First, we looked into the correlational coefficients among variables in Table 3 and found that none of the pairs had a very high correlation (*r*>.90) [53]. Second, we conducted Harman single-factor test using principle component analysis in SPSS 18.0 of International Business Machines Corporation in United Stated. Ten factors were extracted and the first factor in the unrotated solution explained 31%, which is less than 50% [54]. Third, we employed the marker variable technique to test common method bias [55]. We used perceived organizational support as the marker variable. The average correlation among perceived organizational support and those of the principle constructs is *r*=.198. Therefore, common method bias may not be an issue in our study.

**Table 2:** Construct reliability and convergent validity.

Construct and items		Factor loadings	Composite reliability	Average variance extracted	Cronbach alpha
**Instrumental interaction (INI)**					
	INI1	0.8021	0.8125	0.5912	.6547
	INI2	0.7388			
	INI3	0.7644			
**Affective interaction (AI)**					
	AI1	0.7639	0.8051	0.5793	.6369
	AI2	0.7704			
	AI3	0.7489			
**Declarative knowledge (DK)**					
	DK1	0.7566	0.8564	0.5444	.7912
	DK2	0.769			
	DK3	0.7582			
	DK4	0.7022			
	DK5	0.7002			
**Self-efficacy (SE)**					
	SE1	0.7798	0.8876	0.6124	.8419
	SE2	0.7624			
	SE3	0.7873			
	SE4	0.7869			
	SE5	0.796			
**Outcome expectancy (OE)**					
	OE1	0.7635	0.8342	0.6239	.6983
	OE2	0.8345			
	OE3	0.7697			
**Health behaviors (HB)**					
	HB1	0.8094	0.9021	0.5688	.8737
	HB2	0.7335			
	HB3	0.7244			
	HB4	0.7611			
	HB5	0.731			
	HB6	0.7916			
	HB7	0.7232			

**Table 3:** Discriminant validity. The square roots of average variance extracted (AVEs) are in italics.

Constructs	Instrumental interaction	Affective interaction	Declarative knowledge	Self-efficacy	Outcome expectancy	Health behaviors
Instrumental interaction	*0.7681*					
Affective interaction	0.558	*0.7611*				
Declarative knowledge	0.4757	0.4346	*0.7378*			
Self-efficacy	0.4204	0.4217	0.2574	*0.7826*		
Outcome expectancy	0.5193	0.4653	0.5408	0.3536	*0.7899*	
Health behaviors	0.4463	0.403	0.3924	0.39	0.3597	*0.7542*

## Results

### Analysis Results of Hypothesized Model

**Figure 2:**
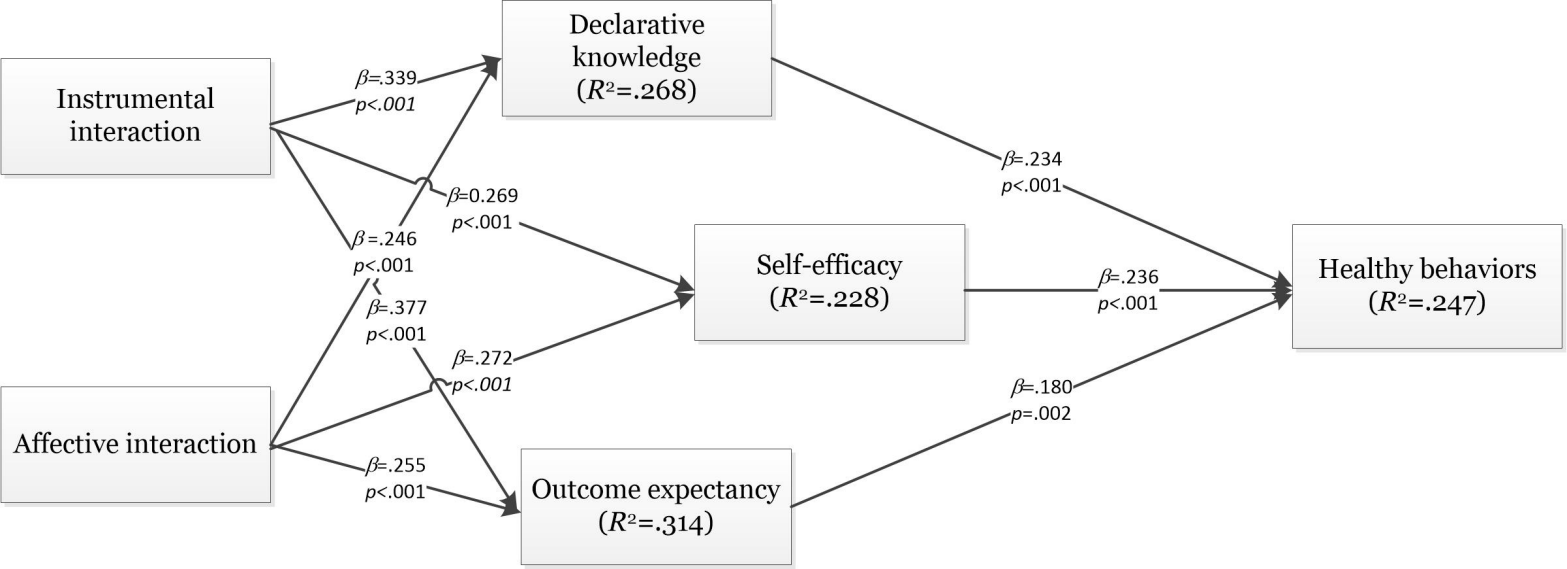
Analysis results of structural model.

PLS with bootstrapping procedure was used to test the hypothesized model. Estimates derived from the PLS analysis were used to test the research hypotheses. The results of the analysis are summarized in Figure 2. The results revealed that DK, SE, and OE significantly influenced consumers’ health behaviors. The significant effect of these 3 constructs demonstrated the explanatory power of social exchange theory. Therefore, H1, H2, and H3 were all supported. With regard to the impact of the interaction between doctors and consumers, the results showed that both types of doctor-consumer interactions significantly affected consumers’ DK, SE, and OE. These results manifested that the interaction between doctors and consumers on social media could increase consumers’ DK of health, enforce their SE of doing healthy lifestyle behaviors, and lead to positive OE of doing healthy lifestyle behaviors. Therefore, H4a, H4b, H5a, H5b, H6a, and H6b were all supported.

### Mediation Analysis of Declarative Knowledge, Self-Efficacy, and Outcome Expectancy

To test the mediation role of DK, SE, and OE, we adopted the bootstrapping technique [56,57]. Compared with traditional methods such as the Baron and Kenny [58] method and the Sobel [59] method, the bootstrapping method can test the indirect effect of independent variables on dependent variables directly and does not require the normal distribution of mediation effect [60]. In this study, the 95% confidence interval of the indirect effects was obtained with 5000 bootstrap resamples. By using the SmartPLS 2.0 M3 [51], we summarize the mediation analysis results in Table 4. According to the results, the indirect effects of instrumental interaction and affective interaction on health behaviors were significant. Therefore, DK, SE, and OE significantly mediated the relationship between doctor-consumer interaction and health interaction. Meanwhile, based on the significance of direct effect, the effect of instrumental interaction on health behaviors was partially mediated by that of DK, SE, and OE, whereas the relationship between affective interaction and health behaviors was fully mediated by that of DK, SE, and OE.

**Table 4:** Mediation analysis using bootstrapping method.

Independent variable	Mediating variable	Dependent variable	Indirect effect			Direct effect			Mediation proportion
			2.5% CI	97.5% CI	Effect value	2.5% CI	97.5% CI	Effect value	
INI^a^	DK^b^	HB^c^	0.0275	0.1311	0.0793	0.0744	0.3096	0.1920	Partial mediation
INI	SE^d^	HB	0.0186	0.1084	0.0635	0.0744	0.3096	0.1920	Partial mediation
INI	OE^e^	HB	0.0159	0.1198	0.0679	0.0744	0.3096	0.1920	Partial mediation
AI^f^	DK	HB	0.0180	0.0972	0.0576	−0.0050	0.2437	0.1220	Full mediation
AI	SE	HB	0.0181	0.1103	0.0642	−0.0050	0.2437	0.1220	Full mediation
AI	OE	HB	0.0057	0.0861	0.0459	−0.0050	0.2437	0.1220	Full mediation

^a^INI: instrumental interaction.

^b^DK: declarative knowledge.

^c^HB: health behaviors.

^d^SE: self-efficacy.

^e^OE: outcome expectancy.

^f^AI: affective interaction.

## Discussion

### Principal Findings

In this paper, we studied the effect of doctor-consumer interaction on social media on consumers’ health behaviors empirically. On the basis of professional-client interaction theory, we divided doctor-consumer interaction into instrumental interaction and affective interaction and conceptualized them in the social media context. In the meantime, depending on social cognitive theory, we proposed 3 variables that mediate the relationship between doctor-consumer interaction on social media and consumers’ health behaviors: DK, SE, and OE. To test our hypotheses, we established a research model by integrating the above theories and developing corresponding measurement instruments. By using the survey method, we collected data from consumers who had the experience of interacting with doctors in China. By analyzing the data, we found that all our hypothetical relationships were supported. Therefore, we can conclude that interacting with doctors on social media can improve consumers’ health behaviors.

Furthermore, we also looked into the mediation effect of the 3 proposed mediators. By using the advanced bootstrapping method, we discovered that the effect of instrumental interaction on health behaviors was partially mediated by DK, SE, and OE, whereas the effect of affective interaction on health behaviors was fully mediated by the above mediators. Therefore, the 3 mediators are adequate to explain the process from instrumental interaction to health behaviors, whereas more potential mediators are needed to be explored for the effect of affective interaction on health behaviors.

### Implications

This study brings a few interesting contributions to theory and practice. From the theoretical perspective, we extend professional-client interaction theory into the social media context by conceptualizing doctor-consumer interaction in social media and dividing it into instrumental and affective interaction. Our empirical study confirms the effectiveness of this extension. Meanwhile, the 2 types of interaction provide a deep insight into understanding the role of doctor-consumer interaction.

Second, we integrate professional-client interaction theory and social cognitive theory in this study. Professional-client interaction theory helps us understand doctor-consumer interaction in the social media context, whereas social cognitive theory points out the underlying working mechanisms of the effect of doctor-consumer interaction. By integrating these 2 theories, we describe a full map of the role of doctor-consumer interaction.

Third, we propose and test 3 working mechanisms of doctor-consumer interaction. DK, SE, and OE are proposed as the working mechanisms based on social cognitive theory. Compared with previous literature, we first consider the role of DK in health behaviors and test all the 3 factors in the social media context. Especially, our mediation analysis uncovered that these 3 mediators fully mediated the effect of affective interaction and partially mediated the effect of instrumental interaction.

From a practical perspective, this study suggests that doctor-consumer interaction can be considered as a natural intervention to change consumers’ health behaviors and then their health status. Therefore, compared with traditional offline health education and promotion activities, health care providers and health educators could pay attention to doctors’ activities on social media. Doctor-consumer interaction guidelines should be developed. Meanwhile, consumers should be encouraged to interact with doctors on social media.

Second, both instrumental and affective interaction could be considered in doctor-consumer interaction on social media. Compared with interaction in the offline context, the role of affective interaction should be highlighted in the social media context. For example, besides providing professional suggestions to consumers, doctors should show their interests on consumers’ health problems and give them enough chances to express their anxiety and confusion.

Finally, the proposed working mechanisms can help evaluate the effectiveness of doctor-consumer interaction. Health care providers and health educators can even refer our measurement scales to check the effects of their interaction with consumers on social media.

### Limitations and Future Work

The results of this study should be interpreted in the light of its limitations. First of all, we have indeed identified several working mechanisms of doctor-consumer interaction; however, our mediation analysis indicates that more working mechanisms await exploration, especially for the instrumental effect. Future studies can consider other mediators and other theoretical perspectives to improve the validity of our research model. Moreover, interaction among consumers about health problems may also influence consumers’ attitude toward health behaviors. Future studies can include both doctor-consumer interaction and consumer-consumer interaction.

Second, the generalizability may be restricted as our sample is restricted to Chinese consumers rather than people from other countries. In China, the two most popular social media platforms are WeChat and Weibo [45], but in other countries, other social media platforms such as Facebook or Twitter are more dominant. Differences between WeChat or Weibo and social media platforms in other countries exist. For example, Twitter is a global microblogging service provider and keeps itself simple, whereas Weibo focuses on China and adds many features in its platform [61]. These differences may make consumers in these 2 platforms behave differently. Future studies may conduct cross-country comparisons to better generalize the results of this study.

Third, our study is a cross-sectional one in which constructs were measured at the same point of time. However, as consumer behavior and social media are both dynamic, the results may change with the passage of time. Therefore, the cross-sectional design may not reflect the dynamics of social media usage. Meanwhile, the time sequence of independent variables, mediators, and dependent variables could not be revealed in a cross-sectional survey. A longitudinal study that collects the data of different variables at different times may help address this issue.

Finally, although the explained variance of health behaviors in our structural model is acceptable, some unexplained variance remains and other relevant factors should be explored. In this study, we applied the social cognitive theory and only considered the personal factors including DK, SE, and OE to explain health behaviors; other situational and environmental factors should be included in future studies. Moreover, other theories such as the health belief model could be applied to understand health behaviors [62].

### Conclusions

This paper contributes to the literature on doctor-patient communication by investigating doctor-consumer interaction on social media. Our study demonstrated the important role of doctor-consumer interaction on social media for consumers’ health behaviors. This result not only implies that social media could be feasible channels to promote consumers’ health behaviors but also reveals that doctors could consider engaging in using social media to interact with consumers for health purposes. The significant mediating role of DK, SE, and OE consists of the working mechanisms of doctor-consumer interaction on social media. Theoretical and practical implications for leveraging social media to promote health behaviors are provided.

## Appendix

Multimedia Appendix 1 Measurement instruments.

## Abbreviations

AI: affective interaction
AVE: average variance extracted
DK: declarative knowledge
HB: health behaviors
INI: instrumental interaction
OE: outcome expectancy
SE: self-efficacy
