# Supplementary material for: The Effect of Doctor-Consumer Interaction on Social Media on Consumers’ Health Behaviors: Cross-Sectional Study
Source: J Med Internet Res. 2018 Feb 28;20(2):e73. doi: 10.2196/jmir.9003 (PMC5852273; doi:10.2196/jmir.9003)
Supplement: Multimedia Appendix 1 [file jmir_v20i2e73_app1.pdf]

Table A1. Measurement instruments

Constructs	Items
Instrumental Interaction	• The doctors who I interact with in social media have skills to solve my problems/concerns<br>• The doctors who I interact with in social media give me required suggestions and information.<br>• My interaction with doctor in social media is about the solution of my health concern.
Affective interaction	• During my interaction with the doctors in social media, the doctors show their interest in my problems/concerns.<br>• During my interaction with the doctors in social media, the doctors demonstrate sufficient devotion to the management of my problems<br>• During my interaction with doctors in social media, the doctors give me emotional support.
Declarative knowledge	*Copyright of this scale is held by Prof. Bernadette Mazurek Melnyk.*
Self-efficacy	• I will be able to achieve most of the goals that I have set for myself.<br>• In general, I think that I can obtain outcomes that are important to me.<br>• I am confident that I can perform effectively on many healthy lifestyle behaviors.<br>• I know how to deal with things in a healthy way that bother me<br>• Compared with other people, I do most healthy behaviors very well
Outcome expectancy	• If I do healthy lifestyle behaviors more than before, I will sleep better.<br>• If I do healthy lifestyle behaviors more than before, I will feel refreshed.<br>• If I do healthy lifestyle behaviors more than before, I would feel

	better mentally.
Healthy behaviors	*Copyright of this scale is held by Prof. Bernadette Mazurek Melnyk.*
